# Conservation and Evolution of Antigenic Determinants of SARS-CoV-2: An Insight for Immune Escape and Vaccine Design

**DOI:** 10.3389/fimmu.2022.832106

**Published:** 2022-04-04

**Authors:** Varun Jaiswal, Hae-Jeung Lee

**Affiliations:** ^1^ Department of Food and Nutrition, College of BioNano Technology, Gachon University, Seongnam-si, South Korea; ^2^ Institute for Aging and Clinical Nutrition Research, Gachon University, Seongnam-si, South Korea; ^3^ Department of Health Sciences and Technology, GAIHST, Gachon University, Incheon, South Korea

**Keywords:** COVID-19, SARS-CoV-2, evolution, immunity, vaccine, mutation, B-cell epitopes, T-cell epitopes

## Abstract

Coronavirus disease 2019 (COVID-19) is the most devastating pandemic of the century, which is still far from over. The remarkable success of severe acute respiratory syndrome coronavirus 2 (SARS-CoV-2) vaccines is the working hope, but the evolving variants are the huge concern that can turn the tide. Potential immune escape mutations (PIEMs) in the past and circulating variants were not studied at large scale (all available data). Hence, the conservation of antigenic determinants (epitopes) was analyzed in all available sequences of SARS-CoV-2 according to time (months), proteins, hosts, and variants. Numerous highly conserved B- and T-cell epitopes were identified in 24 proteins of SARS-CoV-2. A decrease in the conservation of epitopes with time was observed in almost all proteins, which was more rapid in neutralizing epitopes. Delta variant still has the highest PIEM in the circulating strains, which pose threat to the effectiveness of current vaccines. The inclusion of identified, highly conserved, and important epitopes in subunit vaccines can increase vaccine effectiveness against evolving variants. Trends in the conservation of epitopes in different proteins, hosts, and variants with time may also help to inspire the counter measure against the current pandemic.

## Introduction

The origin and initial cases of coronavirus disease 2019 (COVID-19) were reported from Wuhan, China at the end of the year 2019, which was due to a novel coronavirus later named as severe acute respiratory syndrome coronavirus 2 (SARS-CoV-2). The COVID-19 was declared a pandemic on March 11, 2020 by the World Health Organization. The current pandemic has already claimed more than 4.9 million lives from more than 244 million infections globally (1). The primary protective medical intervention against COVID-19 are vaccines, which are found to be effective in different studies and got regulatory approvals, although 100% protection is not reported for any vaccine, and variants arising due to the mutations are concerns for the vaccine effectiveness (2, 3). The emerging variants, reinfection, and infection after vaccination was observed in a significant number of cases, which warrants further research to develop a more effective vaccine for COVID-19 (4, 5). In breakthrough cases, the vaccination decreases complications and death; still, elderly patients with significant comorbidities are at an elevated risk irrespective of vaccination status (6). The fate of the current pandemic would be largely dependent on evolving virus variants that can escape immunity conferred through the vaccination or past infection (7). The studies after the 6 months of second dose of the vaccine found a substantial decrease in humoral immune response (8), and waning immunity was observed in all age groups (9). Waning immunity and evolving variants (mutants) with time might be the serious threat to the current vaccination strategies (9). Mutation in the virus is a normal process that occurs spontaneously, although most of the mutations are expected to be neutral or have minimal effect on the biology of the virus (10). Although few mutations may contribute to virus fitness and adaptation. The minority of mutations are expected to provide the fitness advantage to the virus. Such mutations can alter various virus biology in the host like infectivity, virulence, transmissibility, antigenicity, and protective immune response of the host (10). The mutation in the part important for antigenicity may be considered as the most important mutation for escape variants (11, 12). The efficacy of vaccines was found to be reduced in the evolved variants such the Delta variant (2, 13, 14). The Delta variant was found to be more infectious (15) and considered as the major variant in the deadly second wave in India, which reported more than 25,000 cases of infection and 400 deaths in peak days from the national capital only (13, 16). Although single mutations in epitope can lead to the escape of virus from most of the antibodies (17, 18), a further mutation in the circulating strains/variants may accumulate more escape mutation with time, which can possess a threat to the effectiveness of current vaccines. The study of conservation of antigenic region in the genome is highly required to understand the effectiveness of vaccines in the evolving variants and coin the counter measure accordingly (19).

Fortunately, a large number of epitopes were discovered from the SARS-CoV-2 at a short period of time, which can be utilized for conservation and other studies. Similarly, huge genome sequencing efforts around the world have provided a rich resource of genomic sequences. These resources can be used to calculate the conservation of the different genomic regions, especially the regions that encode epitopes. Earlier conservation analysis in SARS-CoV-2 was conducted on small datasets and not focused on immunity or majorly based on predicted epitopes (20). Known B- and T-cell epitopes were not studied for their conservation in the past, and circulation strains of SARS-CoV-2 can be crucial for the identification of immune escape mutation. Thus, the large-scale analysis of all available genome sequences was carried out to study the conservation of all experimentally known epitopes. The identification of highly conserved epitopes that emerged in the study can encounter the emerging variants for vaccine effectiveness, if used in the vaccine construct. The current analysis also provides the opportunity to visualize the evolution/change happening on the antigenic region of the SARS-CoV-2 during the current pandemic.

## Materials and Methods

All sequences of 27 proteins (Spike, M, N, E, NSP1, NSP2, NSP3, NSP4, NSP5, NSP6, NSP7, NSP8, NSP9, NSP10, NSP11, NSP12, NSP13, NSP14, NSP15, NSP16, NS3, NS6, NS7a, NS7b, NS8, NS9b, and NS9c) along with strain IDs and additional information corresponding to all available SARS-CoV-2 genomes from the Global Initiative on Sharing Avian Influenza Data (GISAID) (21) were extracted. More than 3 million sequences for each 27 proteins were used in this analysis. All partial sequences (less than the known length) and sequences with non-standard amino acids “X” were discarded. The associated information such as country, date of sampling, host, variant, and submitter information were also stored accordingly. Similarly, all known T-cell (1,928) and linear B-cell epitopes (3,923) of SARS-CoV-2 were downloaded from the Immune Epitope Database (IEDB) (22) with associated information. Only unique B- and T-cell epitopes with a positive result in the experimental assay were used in the analysis. Linear neutralizing epitopes of SARS-CoV-2 were also downloaded from the IEDB database (22). These epitopes are the subset of BCE from SARS-CoV-2, which were found to be neutralizing in the *in vivo* studies (human or animal studies). Therefore, neutralizing epitopes can be considered as more important for inducing protective immune responses. Additionally, epitopes from the region that interact with host protein [angiotensin-converting enzyme-2 (ACE2)] to gain entrance into the host cells were also identified and studied for conservation. The co-crystal structure of spike and human ACE2 protein complex (PDB ID: 6M0J) was used to identify all interacting residue in the spike protein with the help of PDBsum (23). BCE from the spike protein, which had any identified interacting residue, was considered as the epitope from the interaction region (EIR), and antibodies to that epitope may potentially inhibit the binding of the host receptor ACE2.Calculation of epitope conservation.

The conservation of each epitope was calculated in the corresponding protein according to the number of strains, variants, months, and hosts. In-house developed PERL scripts were used to organize the data and calculate conservation, and results were saved in CSV files for further analysis. Strain-wise conservation of each epitope was calculated as the ratio of the number of strains in which the epitope was conserved (NSEC) with a number of total strains used in the study [strains in which the epitope was found to be mutated (NSEM) + NSEC] according to **Equation 1**. Similarly, the monthly conservation of each epitope was calculated every month (from December 2019 to September 2021) according to **Equation 2**. Variants-wise conservation of epitopes was calculated for all the important variants (Alpha, Beta, Delta, Eta, Gamma, Kappa Lambda, Iota, and Mu variants) listed in GISAID database (**Equation 3**). Conservation of epitopes was also calculated according to the host in which the sample was isolated (**Equation 4**).


Equation 1 $$EC=\frac{NSEC}{NSEC+NSEM}$$

Equation 2 $$EC_{M}=\frac{NSEC^{M}}{NSEC^{M}+NSEM^{M}}$$

where superscript *M* represents the month of sampling (beginning from December 2019).


Equation 3 $$EC_{V}=\frac{NSEC^{V}}{NSEC^{V}+NSEM^{V}}$$

where superscript *V* represents the variants used in the study.


Equation 4 $$EC_{H}=\frac{NSEC^{H}}{NSEC^{H}+NSEM^{H}}$$

where superscript *H* represents the hosts from sample were used for sequencing.

### Linear Regression in Monthly Conservation of Epitopes

To study the trends of conservation/mutation of all epitopes with time, the linear regression analysis with a best fit line on month-wise conservation of epitopes was carried out separately for B- and T-cell epitopes in each protein. The linear regression model is depicted in matrix form in **Equation 5**. In-house developed Perl script was used to organize data and calculate monthly conservation of epitope for every epitope in the corresponding protein. The regression analysis, plot generation, and other statistical analysis were carried out with R using linear regression and plot functions.

Equation 5: linear regression model in matrix form as


$$\left[\begin{array}{c} EC_{M_{1}}\\ EC_{M_{2}}\\ \vdots \\ EC_{M_{n}} \end{array}\right]=\begin{array}{c} \alpha_{1}+\beta_{1}M_{1},\\ \alpha_{2}+\beta_{2}M_{2}\\ \vdots \\ \alpha_{n}+\beta_{n}M_{n} \end{array}$$

where *α_i_*, *β_i_* are arbitrary constants (intersect and slope) for *i* = 1,2,…, *n*; n is total number of epitope in the particular protein.

### Potential Immune Escape Mutation in Variants

The mutation in the epitope from the spike protein may be the potential immune escape mutation (PIEM), as a mutation in the epitope from (spike protein) most important/only vaccine antigen (in most of the vaccines) may result in reducing immune responses. The variants (Alpha, Beta, Gamma, Delta, Iota, and Mu) with at least 10,000 available genome sequences were studied to calculate the number and percentage of B-cell epitopes with mutation in the spike protein as PIEM. PIEM is calculated every month from the month of origin of the respective variant.


Equation 6 $$PIEM_{MV}\%=\frac{No.of\;BCE\;not\;conserved\;in\;perticulat\;month\;and\;variant\;in\;spike\;protein}{Total\;number\;of\;BCE\;in\;spike\;protein}\times 100$$

### Identification of Epitopes With Both Immune Responses

To identify common BCE and TCE epitopes with neutralizing response, set analysis was carried out using the list of B-cell, T-cell, and neutralizing epitopes. Common epitopes known to be conserved in 99% strains were selected as common conserved epitopes with neutralizing immune response (CCN) and suggested as subunit vaccine candidates for vaccine design. InteractiVenn was used to perform Venn analysis on sets comprised of BCE, TCE, and neutralizing epitopes (24).

## Results

### Conservation of Epitopes

**Figure 1:**
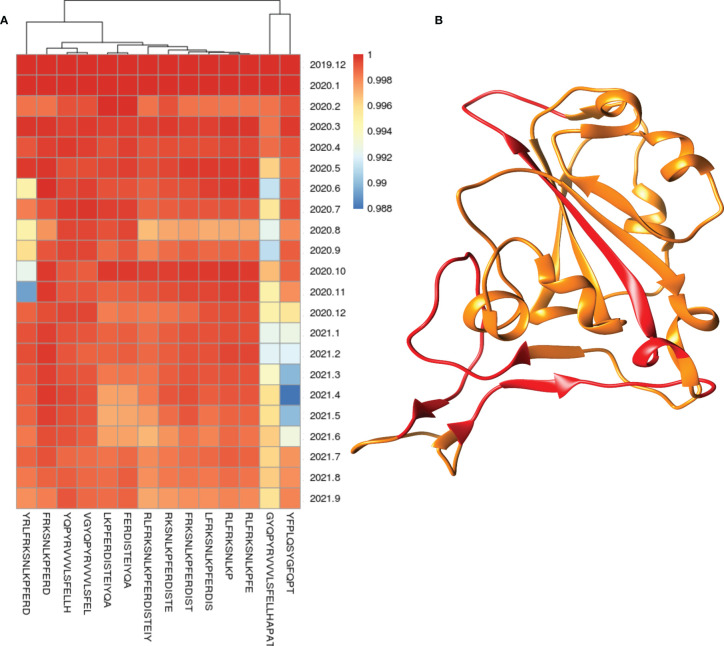
Heatmap of conservation of highly conserved (>99% strains) epitopes from the region which interact with host receptor **(A)** and location of these highly conserved epitopes (shown in red color) in the spike protein receptor-binding domain **(B)**.

Conservation of each epitope was calculated according to the total number and percentage in all strains, variants, host, and month of sampling. The protein-wise conservation of all epitopes is provided in the excel tables (**Tables X1**–**X47**). Total 24 proteins (Spike, M, N, E, NSP1, NSP2, NSP3, NSP4, NSP5, NSP6, NSP7, NSP8, NSP9, NSP10, NSP11, NSP12, NSP13, NSP14, NSP15, NSP16, NS3, NS6, NS7a, NS7b, and NS8) from SARS-CoV-2 were studied for the conservation of epitopes, and 11 proteins were found to have more than 100 BCE and/or TCE (**Tables X1**–**X47**). The highest number of BCE and TCE was found to be present on spike protein (1,002 BCE and 663 TCE) followed by N protein (693 epitopes) in the case of BCE and NSP3 protein (258 epitopes) in the case of TCE (**Tables X1**–**X47**). Most BCE and TCE were found to be conserved in the respective proteins from most of the strains. Conversely, no epitope was found to be conserved in all strains used in the study (**Tables X1**–**X47**). The percentage of the epitopes found to be conserved in 99% or more strains in the proteins (with at least 100 known epitopes) ranges from 19.4% to 95.7% and 55.8% to 94.2% in the case of BCE and TCE, respectively (**Table 1**). N and spike proteins had <70% of epitopes conserved in 99% or more strains in both BCE and TCE (**Table 1**). Total 106 epitopes were identified from the region in spike protein that interacts with host receptor (ACE2) (**Supplementary Table S1** and **Supplementary Figure S1**). A total of 15 epitopes (out of 106) were found to be conserved >99% of strains. Conservation of these epitopes from the month of origin (December 2019) is shown through heatmap (**Figure 1A**), and the location of these highly conserved epitopes is shown in the spike protein receptor-binding domain (**Figure 1B**).

**Table 1:** Highly conserved epitopes in different proteins from SARS-CoV-2.

Sr. No.	Protein Name	Type of epitope	Total no. of epitopes	Epitopes conserved in 99% of strains
1	Spike	BCE	1002	530
		TCE	663	443
2	N	BCE	371	162
		TCE	206	115
3	M	BCE	119	114
		TCE	139	131
4	NSP3	BCE	693	586
		TCE	258	223
5	NSP12	BCE	262	245
		TCE	120	109
6	NSP13	BCE	144	116
7	NSP14	BCE	142	120
8	NSP15	BCE	115	107
9	NS3	BCE	103	20
10	NSP2	BCE	206	159
11	NSP4	BCE	142	114

### Linear Regression in Monthly Conservation of Epitopes Trends

The conservation of both BCE and TCE is found to be decreasing with time according to monthly conservation data of epitopes. Conservation of both BCE and TCE from the spike and N protein is found to be decreased more rapidly among all studied proteins (with more than 50 epitopes) of SARS-CoV-2 according to linear regression (**Supplementary Table S2** and **Supplementary Figures S2**–**S25**). The regression lines were drawn for B-cell, T-cell, EIR, and neutralizing epitopes in the same graph from the monthly conservation data of spike protein to visualize and compare the slope (decrease in the conservation) of each epitope type (**Figure 2**). Among all the epitopes, the highest decreasing slope of the conservation was observed in the case of neutralizing epitopes in linear regression line followed by EIR epitopes (**Figure 2**).

**Figure 2:**
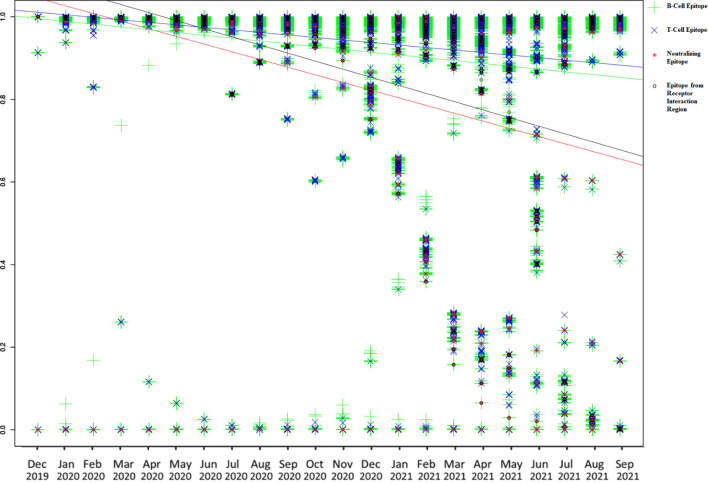
Graphical representation of monthly conservation and linear regression analysis of B-cell, T-cell, EIR, and neutralizing epitopes to demonstrate the trend of conservation. Blue line color is for T-cell epitopes, the yellow line is for B-cell epitopes, black line is for EIR, and the red line is for neutralizing epitopes.

### Potential Immune Escape Mutation in Variants

**Figure 3:**
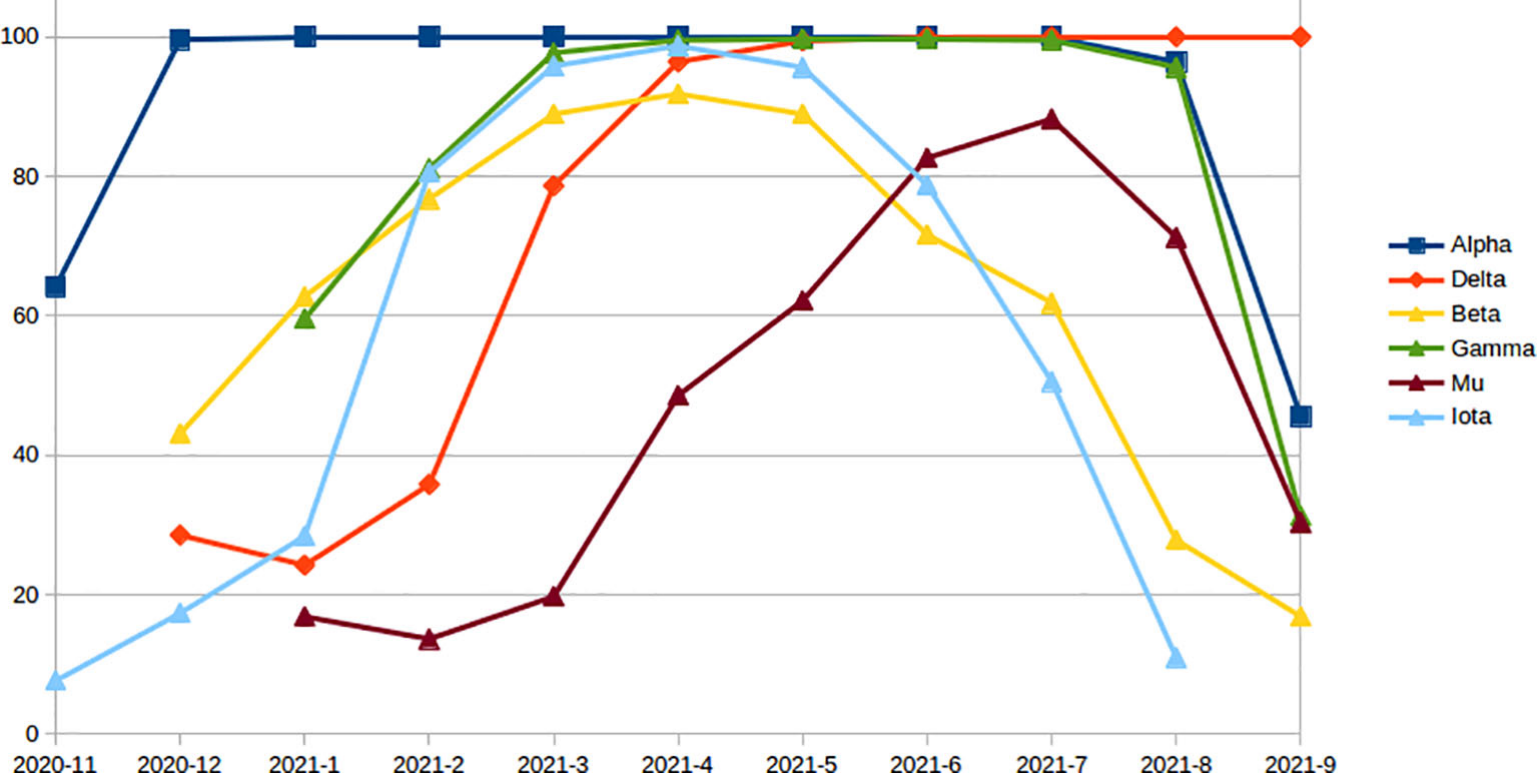
Monthly percentage of epitopes with PEM in different variants since the first reporting month.

The number and percent of PIEM were calculated on important SARS-CoV-2 variants every month until September 2021, since the first reporting month of the variants. In all the variants, the number of escape mutations was found to increase with time to achieve the peak value, and then, a decreasing trend was observed (in Alpha, Beta, Gamma, Iota, and Mu) except for the Delta variant (**Figure 3**). The Delta variant still has a peak value of 100% (**Figure 3**), i.e., for the month of September 2021. The second highest PIEM was still present in the circulating strain of Alpha followed by Gamma and Mu variants (**Figure 3**).

### Conserved Common Epitopes

A total of 187 epitopes were found to be common in both B- and T-cell epitope lists, i.e., these epitopes were known to provide both B- and T-cell immune responses in the literature (**Supplementary Figure S26**). Six out of 187 common epitopes were found to be common with neutralizing epitopes, and out of them, three neutralizing epitopes were found to be highly conserved in SARS-CoV-2 (>95% strains) and proposed as conserved common neutralizing epitopes (CCN) (**Table 2**).

**Table 2:** Conservation of CCN epitopes in all strains of SARS-CoV-2.

Sr. No.	Epitope sequence	Epitope ID (IEDB)	Epitope conservation	Immune response		
				B-cell	T-cell	Neutralizing
1	TFKCYGVSPTKLNDL	1310841	0.998	✔	✔	✔
2	LNEVAKNLNESLIDLQELGK	1309518	0.984	✔	✔	✔
3	YLTPGDSSSGWTAGAAAYYV	1391530	0.965	✔	✔	✔
4	QRNFYEPQIITTDNT	1310750	0.644	✔	✔	✔
5	IYQAGSTPCNGVEGFNCYFP	1383172	0.615	✔	✔	✔
6	VNLTTRTQLPPAYTN	1310909	0.632	✔	✔	✔

## Discussion

Vaccination is the main arsenal against the COVID-19, which seems to be highly effective, especially preventing severe disease almost throughout the globe (25, 26). However, reduced vaccine efficacy against evolving variants (3), reinfection, and breakthrough infection with different variants may pose a serious threat to the current vaccines and vaccination strategies (27).

The variation in the antigenic determinants (epitopes) in the viruses may be a crucial factor for the reduction in protective immunity conferred through vaccines. The mismatch between vaccine antigen and circulating strain of the virus may result in ineffective vaccination (28). Considering the importance of epitopes conservation, the current study was designed and conducted on all available epitope and genomic data of SARS-CoV-2. Conservation of all experimentally known epitopes was calculated on available genomic data sets in the current research. No epitope was found to be conserved in all strains used in the study, but numerous highly conserved BCE and TCE were identified in the corresponding proteins of SARS-CoV-2 (**Table 1** and **Tables X1**–**X47**). Most of the epitopes were found to be highly conserved (**Table 1**), which supports the low overall variation present in the SARS-CoV-2 genomes (29). These highly conserved epitopes seem to be minimally affected from the virus adaptation of immune escape. These epitopes (both BCE and TCE) can be included while carrying out subunit vaccine development to design broad spectrum vaccines against the SARS-CoV-2. Optimized combination of these highly conserved epitopes can also be designed and used for the development of broad spectrum vaccines like multimeric-001, which was designed as a universal Influenza vaccine with highly conserved epitopes (19, 30). The monthly conservation study coupled with linear regression analysis revealed that the conservation of B- and T-cell epitopes has been slightly but gradually decreasing with time in almost all the proteins (**Supplementary Table S2** and **Supplementary Figures S2**–**S25**), which may be the reason for evolving potential escape mutations (vaccine resistance variants). Reduction in the conservation of BCE is more as compared to that of TCE in the spike protein (**Figure 2** and **Supplementary Table S2**), which may suggest the more importance of B-cell epitopes over TCE against the virus, albeit slightly. However, the immune recognition mechanisms of BCE and TCE are different, so the comparison only through a reduction in conservation may not justify the importance of BCE over TCE. Similarly, the higher negative slope compared to other proteins was observed in the case of Spike and N protein (**Supplementary Table S2**), which also highlights more host immune pressure on these proteins and, subsequently, their importance in the protective immune response. As expected, the maximum decrease in the conservation with time was observed in the epitopes known to be neutralizing in the *in vivo* experiments in animals or humans (**Figure 2**). These neutralizing epitopes are considered to be more important for eliciting the protective immune responses. The high decreasing trend in the conservation of antigenic regions of neutralizing immune epitopes indicates the adaptation of virus against the human immune system with time. It could be one of the prominent reasons for reduced effectiveness against the COVID-19 especially in emerging variants (5). These variants of concern are considered as the potential reason for the escape from the protection conferred through the vaccines (2, 14, 25). Potential immune escape mutation present in these variants with time can provide important insight regarding possible mechanisms (31). Similarly, a high negative slope (compare to all BCE) was also observed in the epitopes from the receptor-binding region, which indicates that more mutations have occurred with time in these epitopes as compared to all BCE. It may suggest the importance of these epitopes from the receptor-binding region in protective immunity (32). Furthermore, monthly conservation analysis in variants revealed the initial increase in PIEM in all the variants with time to attain the peak value (such as Alpha, Beta, Gamma, Delta, Iota, and Mu). Except for the Delta variant, other variants showed the reduction in PIEM after the peak point (**Figure 3**). The percentage of PIEM was at its peak value (until September 2021) in the case of Delta variants (**Figure 3**). This illustrates the fact that circulating strains of the Delta variant had potential escape mutations in all known epitopes, which may further reduce the vaccine effectivity in near future. Conserved epitope-based subunit vaccines that activate both humoral and cellular response can be better vaccines for humans in similar viral diseases such as Influenza (33, 34). CCN epitopes identified in the study are proposed as prospective candidates for subunit vaccine development, as these conserved epitopes can induce both humoral and cellular arms of the immune response (**Table 2**). *In vivo* known neutralizing property and the high conservation of CCN epitopes also support their effectiveness against evolving variants, which can be used as the prospective vaccine candidate for subunit vaccine design. Antigen-based diagnosis of COVID-19 can also be affected through epitope mutation in the virus. Mutation can also suppress the detection of SARS-CoV-2 in diagnosis (35). The enzyme-linked immunosorbent assays (ELISAs) and lateral flow assays (LFAs) are the commonly used immunoassays for diagnosis of SARS-CoV-2, which mostly detect antibodies against Spike and N protein. Mutation in the epitopes corresponding to these antibodies would be important to study the effectiveness of antibodies-based detection. Currently, limited information is available for these epitopes; hence, studies may be designed and conducted in the near future with the availability of information to study the mutation in the epitopes that are the target for antibodies used in the SARS-CoV-2 diagnosis. Importantly, in the current study, the information of antigen processing and HLA restriction is not considered in TCE analysis. HLA presentation can be responsible for different immune responses in the different populations that have differences in the HLA frequency. This limitation may be considered especially if a specific population would be considered in the further study.

Considering the fact that more than 244 million cases of infection have already been reported and still there are more than 18 million active cases present worldwide (1), the current analysis is based on the available sequenced portion (~3.5 million genome sequences) of the total infection; therefore, the number of actual PIEM and reduction in the conservation of epitopes may be higher than the calculated in the current analysis. Nevertheless, this study provides the clear idea about the trends of mutation and conservation of different types of epitopes in different proteins, variants, and host with time, thereby helping to understand the current scenario and pave the path for further appropriate therapeutic interventions to manage the ongoing pandemic.

## Conclusions

The conservation of epitopes is gradually decreasing within the circulating strains every month since the origin of the SARS-CoV-2. Mutations are emerging more rapidly in more important epitopes for immunity, such as neutralizing epitopes in monthly circulating strains, which suggest viral adaptation against the host immunity with time. Potential immune escape mutation, according to epitope number, is found to be still in its peak value (equal to total known epitopes in the spike protein) for the Delta variant in the circulating strains. Highly conserved epitopes from the spike and other proteins were identified in the study, which may be used in vaccine construct to design a more effective vaccine against SARS-CoV-2 and evolving variants.

## Data Availability Statement

The datasets presented in this study can be found in online repositories. The names of the repository/repositories and accession number(s) can be found in the article/**Supplementary Material**

## Author Contributions

Conceptualization, VJ and HJL. Methodology, VJ Formal analysis, VJ Investigation, VJ Writing—original draft preparation, VJ. Writing—review and editing, HJL. Visualization, VJ. Supervision, HJL. Funding acquisition, HJL. All authors have read and agreed to the published version of the manuscript. All authors contributed to the article and approved the submitted version.

## Funding

This work was supported by the “Cooperative Research Program of the Center for Companion Animal Research (Project No. PJ01398402)” of the Rural Development Administration, Republic of Korea.

## Conflict of Interest

The authors declare that the research was conducted in the absence of any commercial or financial relationships that could be construed as a potential conflict of interest.

## Publisher’s Note

All claims expressed in this article are solely those of the authors and do not necessarily represent those of their affiliated organizations, or those of the publisher, the editors and the reviewers. Any product that may be evaluated in this article, or claim that may be made by its manufacturer, is not guaranteed or endorsed by the publisher.
