# Supplementary material for: Conservation and Evolution of Antigenic Determinants of SARS-CoV-2: An Insight for Immune Escape and Vaccine Design
Source: Front Immunol. 2022 Apr 4;13:832106. doi: 10.3389/fimmu.2022.832106 (PMC9014086; doi:10.3389/fimmu.2022.832106)
Supplement: Supplementary file 1 [file DataSheet_1.pdf]

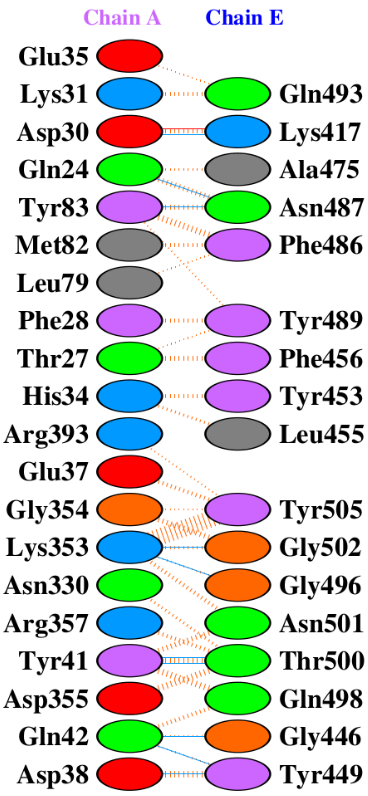

Figure S1: Interacting residues present in human ACE-2 protein (chain A) participated in direct interaction with residues from SARS-CoV-2 spike protein (chain E).

Table S1: B-cell epitopes present at host receptor interacting region of spike protein.

Sr. No.	Epitope Sequence	Start position	End position
1	AGSTPCNGVEGFNCY	474	489
2	NCYFPLQSYGFQPTN	486	501
3	NGVEGFNCYFPLQSY	480	495
4	RKSNLKPFERDISTE	456	471
5	YLYRLFRKSNLKPFE	450	465
6	EGFNCYFPLQSYGFQPTNGV	483	503
7	FERDISTEIYQAGSTPCNGV	463	483
8	GYQPYRVVVLSFELLHAPAT	503	523
9	IAWNSNNLDSKVGGNYNYLY	433	453
10	KVGGNYNYLYRLFRKSNLKP	443	463
11	QAGSTPCNGVEGFNCYFPLQ	473	493
12	QTGKIADYNYKLPDDFTGCV	413	433
13	RLFRKSNLKPFERDISTEIY	453	473
14	SYGFQPTNGVGYQPYRVVVL	493	513
15	EIYQAGSTPCNGVEG	470	485
16	EVRQIAPGQTGKIAD	405	420
17	FRKSNLKPFERDIST	455	470
18	LDSKVGGNYNYLYRL	440	455
19	LKPFERDISTEIYQA	460	475

20	NGVGYQPYRVVVLSF	500	515
21	GYQP	503	507
22	NSNNLDSKVGGN	436	448
23	PCNGVEGFNC	478	488
24	QTGKIADYNYKLPD	413	427
25	RLFRKSNLKP	453	463
26	VRQIAPGQTGKIADYNYKLPDDFTGCVIA	406	435
27	ERDISTEIYQAGSTP	464	479
28	NSNNLDSKVGGNYNY	436	451
29	QIAPGQTGKIADYNY	408	423
30	STPCNGVEGFNCYFP	476	491
31	YFPLQSYGFQPTNGV	488	503
32	YNYLYRLFRKSNLKP	448	463
33	YQAGSTPCNGVEGFN	472	487
34	YQPYRVVVLSFELLH	504	519
35	YRLFRKSNLKPFERD	452	467
36	CNGVEGFNCYFPLQS	479	494
37	CYFPLQSYGFQPTNGVGYQPY	487	508
38	EGFNCYFPLQ	483	493
39	EGFNCYFPLQSYGFQPTNGVGYQPYRVVVLSFELL	483	518
40	NLDSKVGGNYNYLYRLFRKSN	439	460
41	SYGFQPTNGVGYQPY	493	508
42	IRGDEVRQIAPGQTGKIADYNYKLPDDFTG	401	431
43	LDSKVGGNYNYLYRLFRKSNLKPF	440	464
44	NGVEGFNCYFPLQSYGFQPTNGVGYQPYRV	480	510
45	AGSTPCNGVEGFNCYFPLQSYGFQP	474	499
46	LFRKSNLKPFERDIS	454	469
47	NNLDSKVGGNYNYLYR	438	454
48	YGFQPTNGVGYQPYR	494	509
49	TEIYQAGST	469	478
50	DISTEIYQAGSTPCNGVEGFNCY	466	489
51	DISTEIYQAGSTPCNGVEGFNCYFPLQSYGFQPTNGVGYQPYRVVVL	466	513
52	IYQAGSTPCNGVEGFNCYFPLQSY	471	495
53	LKPFERDISTEIYQAGSTPCNGVEGFNCYFPLQ	460	493
54	CNGVEGFNCYFPLQSYGFQP	479	499
55	DEVRQIAPGQTGKIAD	404	420
56	EVRQIAPGQTGKIADY	405	421
57	GDEVRQIAPGQTGKIA	403	419
58	GDEVRQIAPGQTGKIADY	403	421
59	IAPGQTGKIADYNYKL	409	425
60	IYQAGSTPCNGVEGFNCYFP	471	491
61	NYLYRLFRKSNLKPFERDIS	449	469
62	AWNSNNLDSKVG	434	446
63	DSKVGGNYNYLY	441	453
64	EIYQAGSTPCNG	470	482
65	FERDISTEIYQA	463	475
66	FQPTNGVGYQPY	496	508
67	FRKSNLKPFERD	455	467
68	GFQPTNGVGYQP	495	507
69	GSTPCNGVEGFN	475	487
70	GVEGFNCYFPLQ	481	493
71	ISTEIYQAGSTP	467	479
72	IYQAGSTPCNGVEGFNCY	471	489
73	KIADYNYKLPDD	416	428
74	LDSKVGGNYNYL	440	452
75	NGVEGFNCYFPL	480	492
76	PLQSYGFQPTNG	490	502

77	QPTNGVGYQPYR	497	509
78	QSYGFQPTNGVG	492	504
79	RDISTEIYQAGS	465	477
80	RLFRKSNLKPFE	453	465
81	SKVGGNYNYLYR	442	454
82	SNNLDSKVGGNY	437	449
83	STPCNGVEGFNC	476	488
84	SYGFQPTNGVGY	493	505
85	TPCNGVEGFNCY	477	489
86	VEGFNCYFPLQS	482	494
87	WNSNNLDSKVGG	435	447
88	YFPLQSYGFQPT	488	500
89	YQAGSTPCNGVE	472	484
90	NNLDSKVGGNYNYLY	438	453
91	SKVGGNYNYLYRLFR	442	457
92	TGKIADYNYKLPDDF	414	429
93	VEGFNCYFPLQSYGF	482	497
94	VGYQPYRVVVLSFEL	502	517
95	VRQIAPGQTGKIADY	406	421
96	EGFNCYFPLQSYGFQPTNGVGYQPY	483	508
97	EIYQAGSTPCNGVEGFNCYFPLQSYGFQPTN	470	501
98	FRKSNLKPFERDISTEIYQAGSTPCNGVEG	455	485
99	GDEVRQIAPGQTGKIADYNYKLP	403	426
100	IRGDEVRQIAPGQTGKIADYNYK	401	424
101	NLDSKVGGNYNYLYRLFRKSNLKPFERDIST	439	470
102	YRLFRKSNLKPFERDISTEIYQAGS	452	477
103	CVIAWNSNNLDSKVGGNYNYLYRLFRKSNL	431	461
104	GVEGFNCYFPLQSYGFQPTNGVGYQPYRVV	481	511
105	KPFERDISTEIYQAGSTPCNGVEGF	461	486
106	VRQIAPGQTGKIADYNYKLPDDFTGCVIAW	406	436

Table S2: Slope and intersect of linear regression line in monthly conservation of epitope in the proteins with at least 50 known epitopes (graphical depiction is provided in Figure S2-S25).

Sr. No.	Protein Name	Type of Epitope	Total no. of Epitopes	Slope	Intersect
1	Spike	BCE	1002	-0.006781	1.014
		TCE	663	-0.006089	1.017
2	N	BCE	371	-0.006796	1.014
		TCE	206	-0.003151	0.988
3	M	BCE	119	-0.001293	1.006
		TCE	139	-0.001395	0.993
4	NS3	BCE	103	-0.004252	1.013
		TCE	97	-0.001835	0.987
5	NSP3	BCE	693	-0.001199	1.003
		TCE	258	-0.000661	1.000
6	NSP12	BCE	262	-0.001199	1.003
		TCE	120	-0.001835	0.987
7	NSP13	BCE	144	-0.000587	0.999
8	NSP14	BCE	142	-0.000658	.0958
9	NSP15	BCE	115	-0.000188	0.997
10	NSP2	BCE	206	-0.000047	0.990

11	NSP4	BCE	142	-0.002067	0.997
		TCE	73	-0.002057	1.011
12	NS8	BCE	77	-0.003502	0.994
13	NSP1	BCE	70	-0.000131	0.997
14	NSP5	BCE	70	-0.000078	0.997
15	NSP6	BCE	62	-0.004015	1.018
16	NSP8	BCE	56	-0.000128	0.997
17	NSP16	BCE	88	-0.000225	0.985

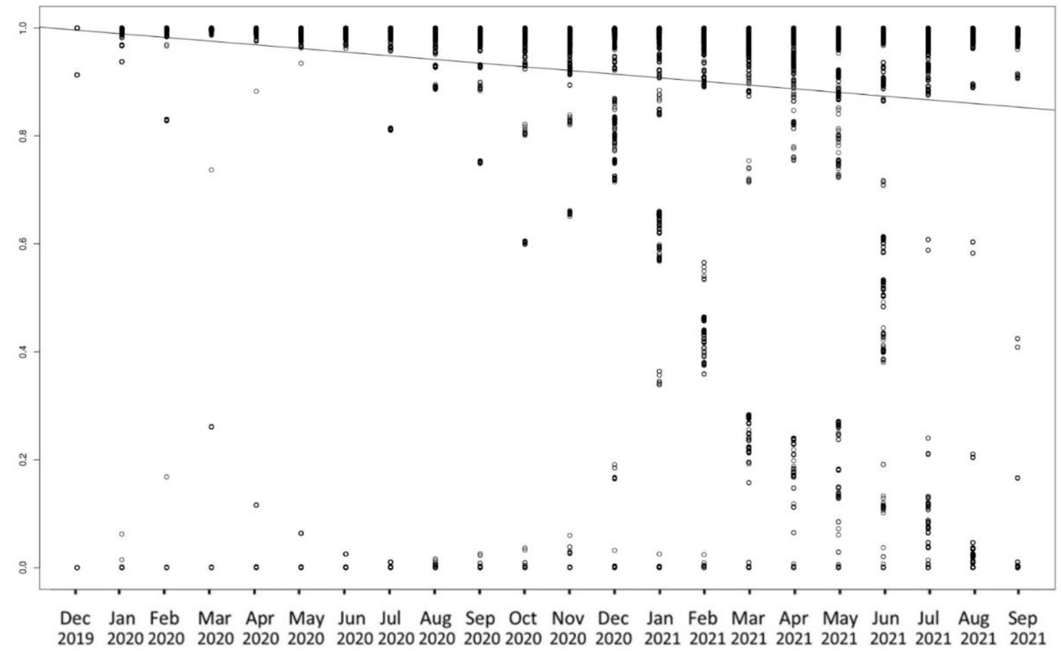

Figure S2: Graph showing linear regression line in monthly conservation of B-cell epitopes (BCE) in the Spike protein.

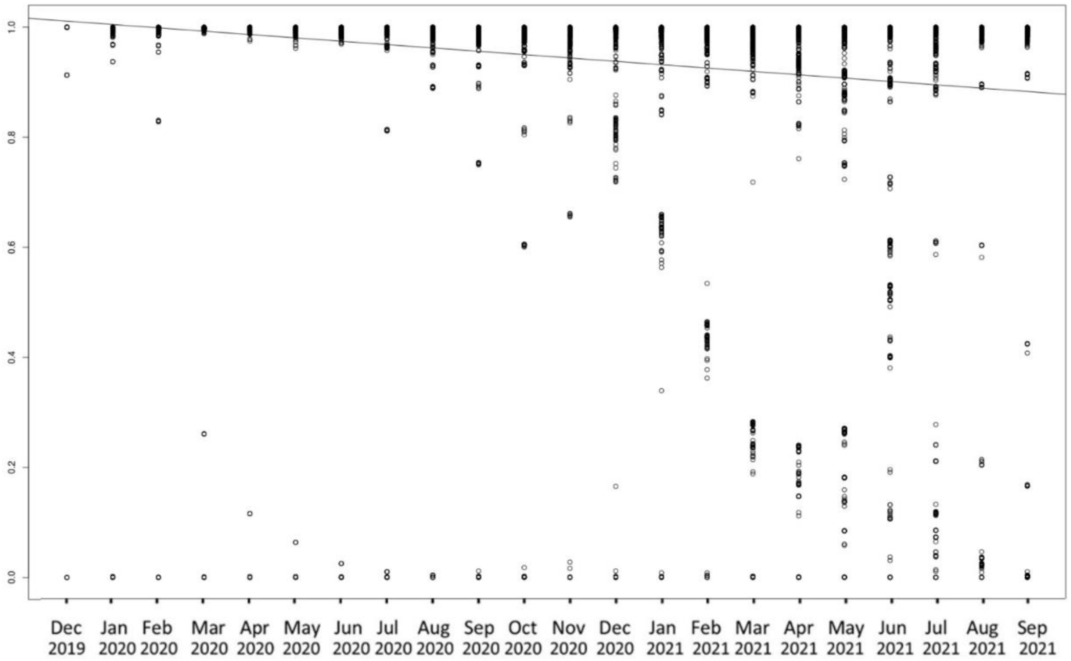

Figure S3: Graph showing linear regression line in monthly conservation of T-cell epitopes (TCE) in the Spike protein.

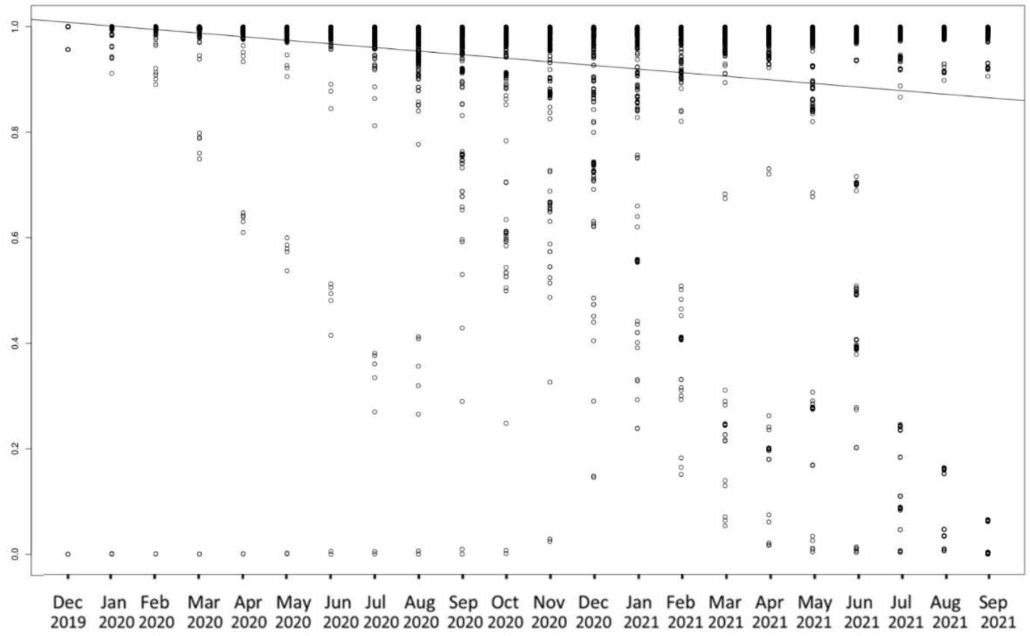

Figure S4: Graph showing linear regression line in monthly conservation of epitopes (BCE) in the N protein.

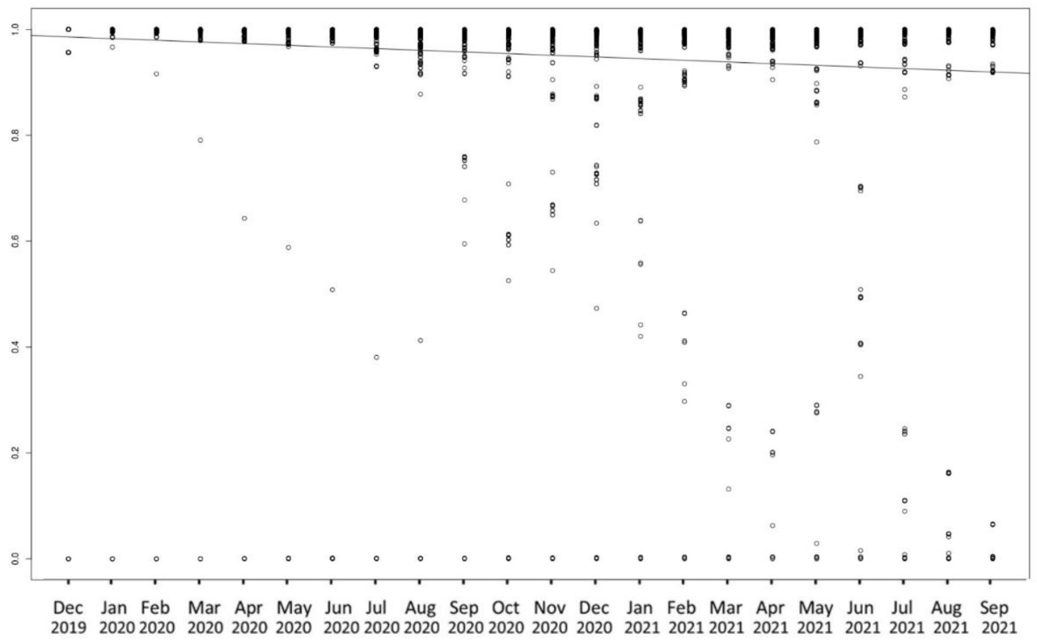

Figure S5: Graph showing linear regression line in monthly conservation of epitopes (TCE) in the N protein.

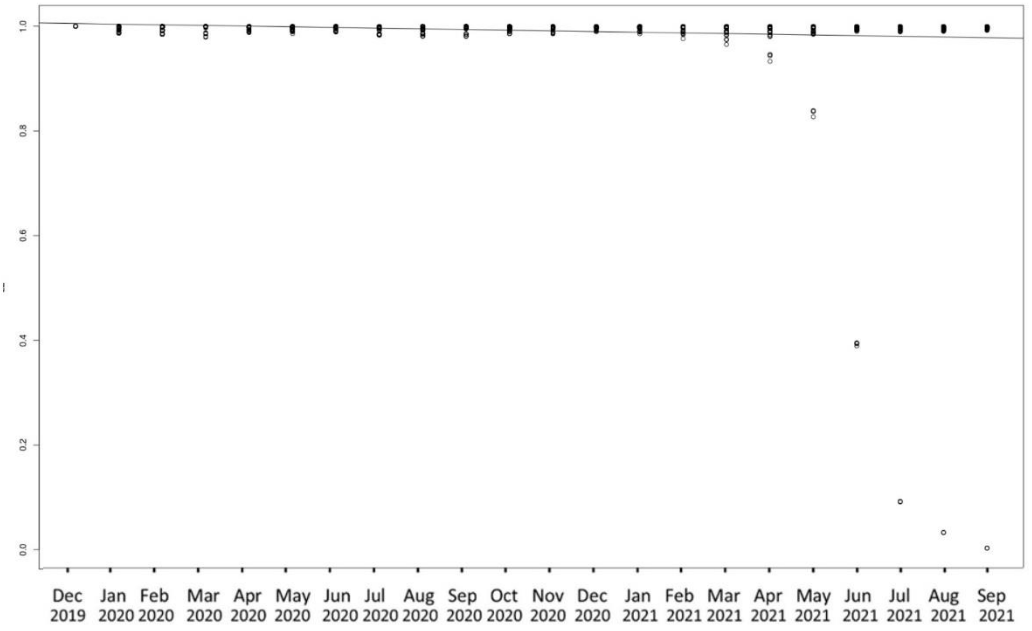

Figure S6: Graph showing linear regression line in monthly conservation of epitopes (BCE) in the M protein.

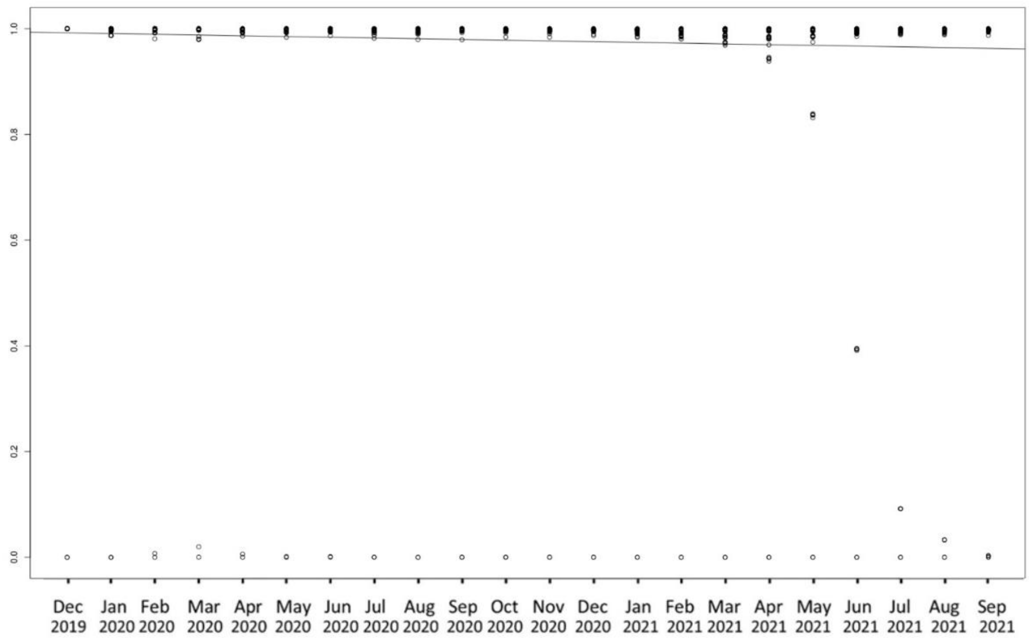

Figure S7: Graph showing linear regression line in monthly conservation of epitopes (TCE) in the M protein.

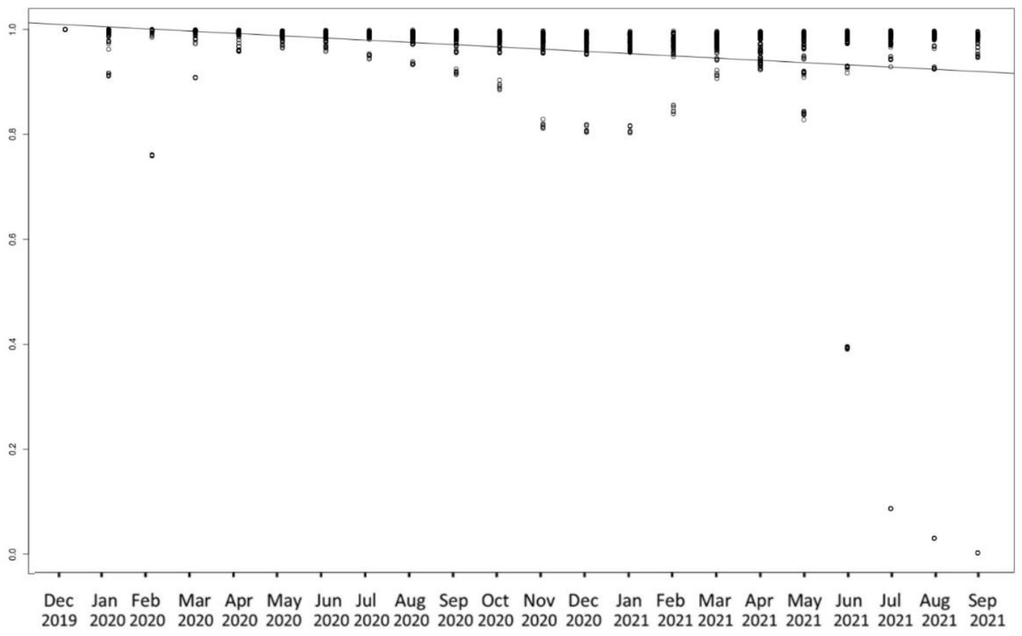

Figure S8: Graph showing linear regression line in monthly conservation of epitopes (BCE) in the NS3 protein.

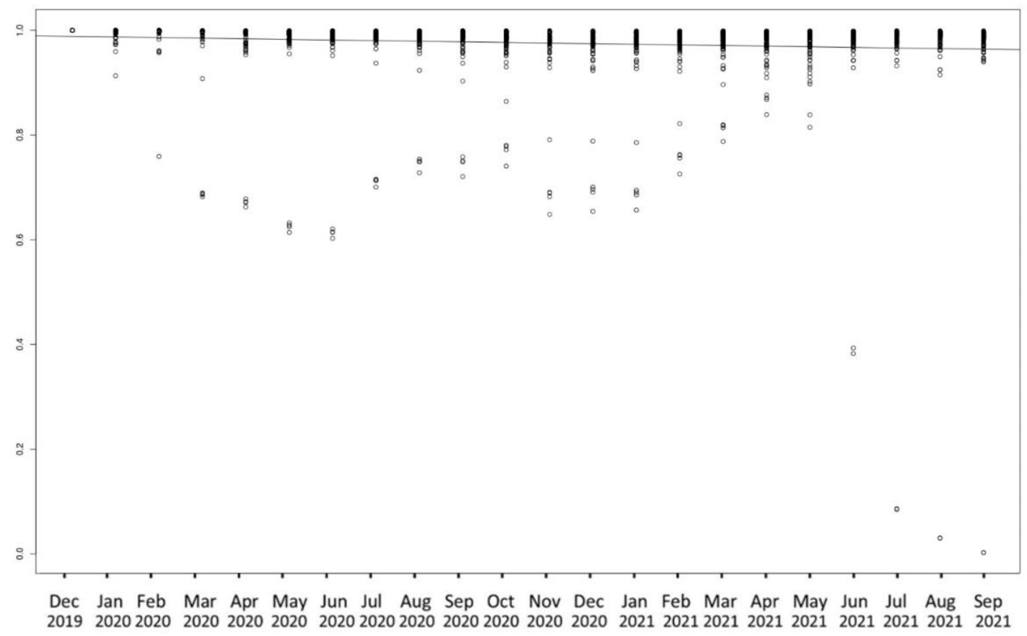

Figure S9: Graph showing linear regression line in monthly conservation of epitopes (TCE) in the NS3 protein.

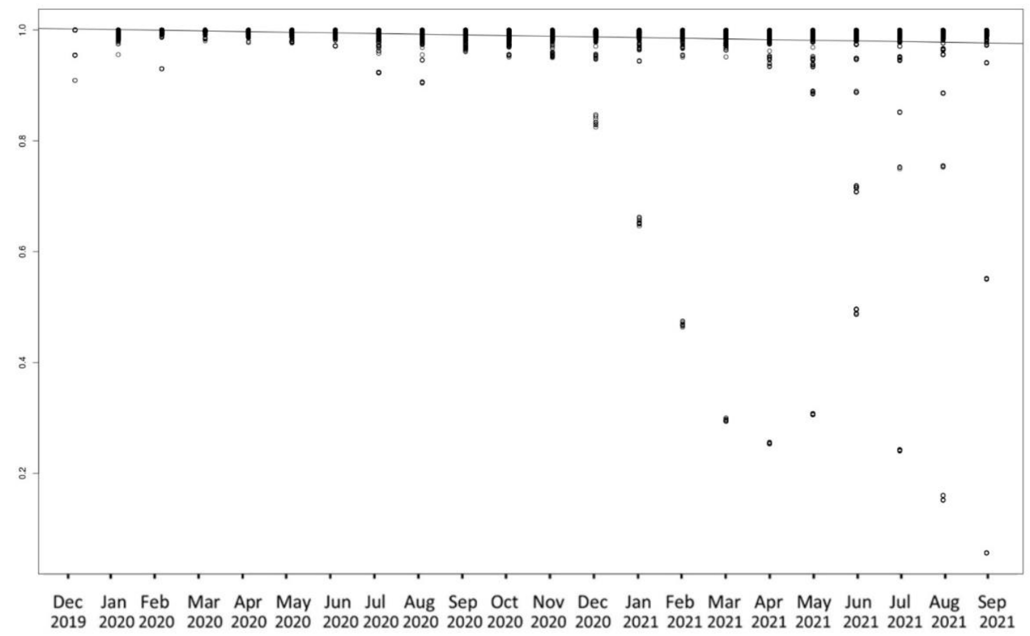

Figure S10: Graph showing linear regression line in monthly conservation of epitopes (BCE) in the NSP3 protein.

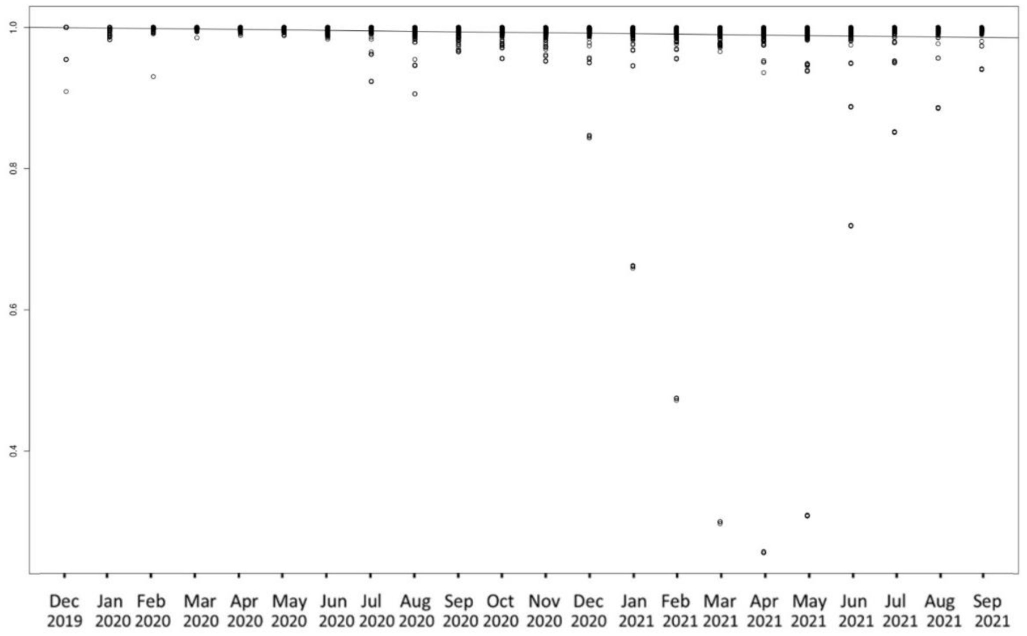

Figure S11: Graph showing linear regression line in monthly conservation of epitopes (TCE) in the NSP3 protein.

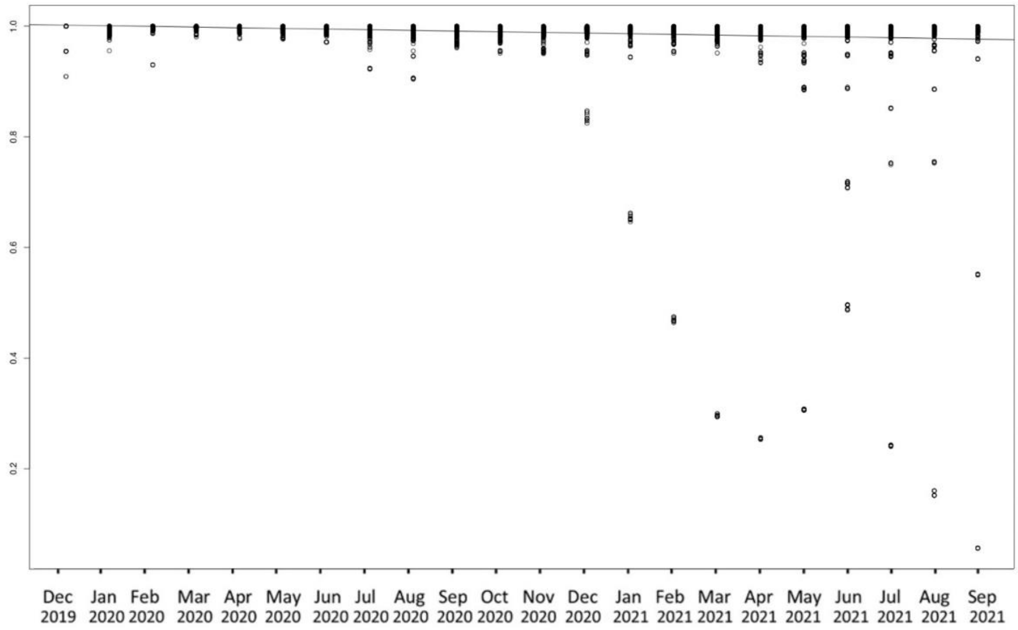

Figure S12: Graph showing linear regression line in monthly conservation of epitopes (BCE) in the NSP12 protein.

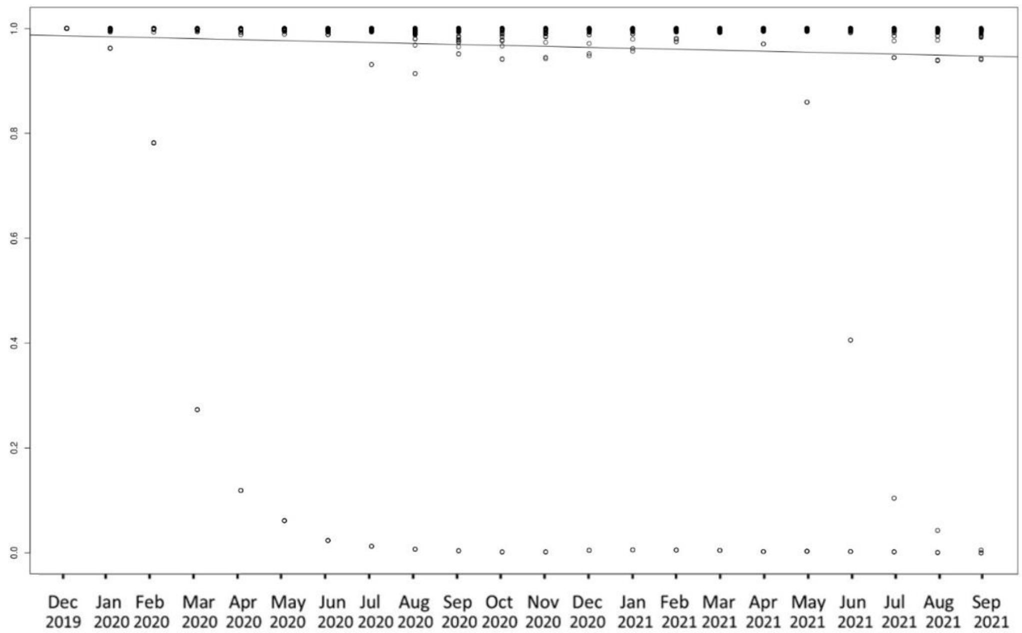

Figure S13: Graph showing linear regression line in monthly conservation of epitopes (TCE) in the NSP12 protein.

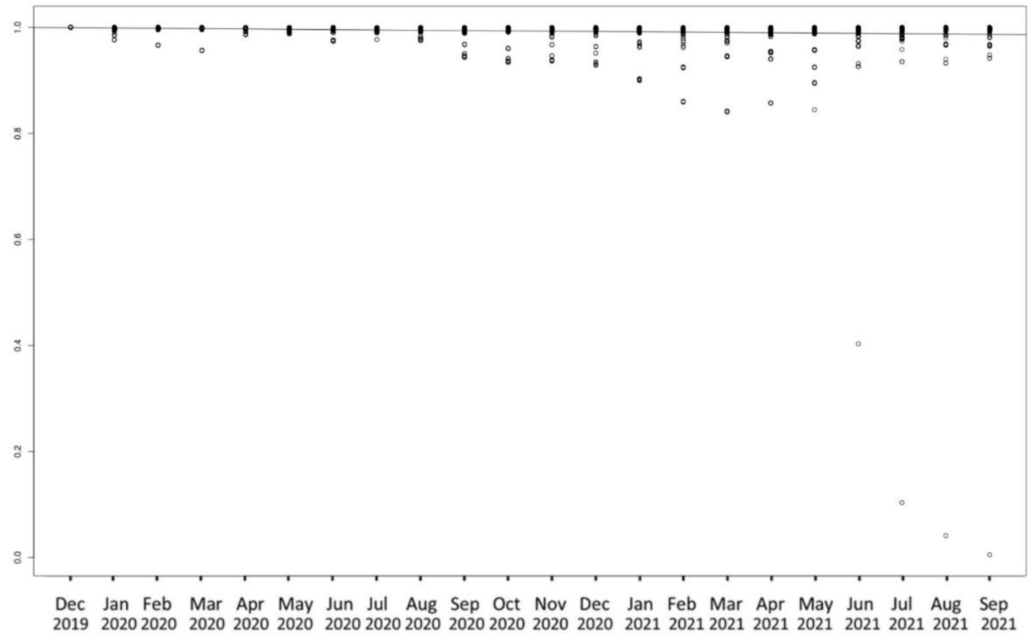

Figure S14: Graph showing linear regression line in monthly conservation of epitopes (BCE) in the NSP13 protein.

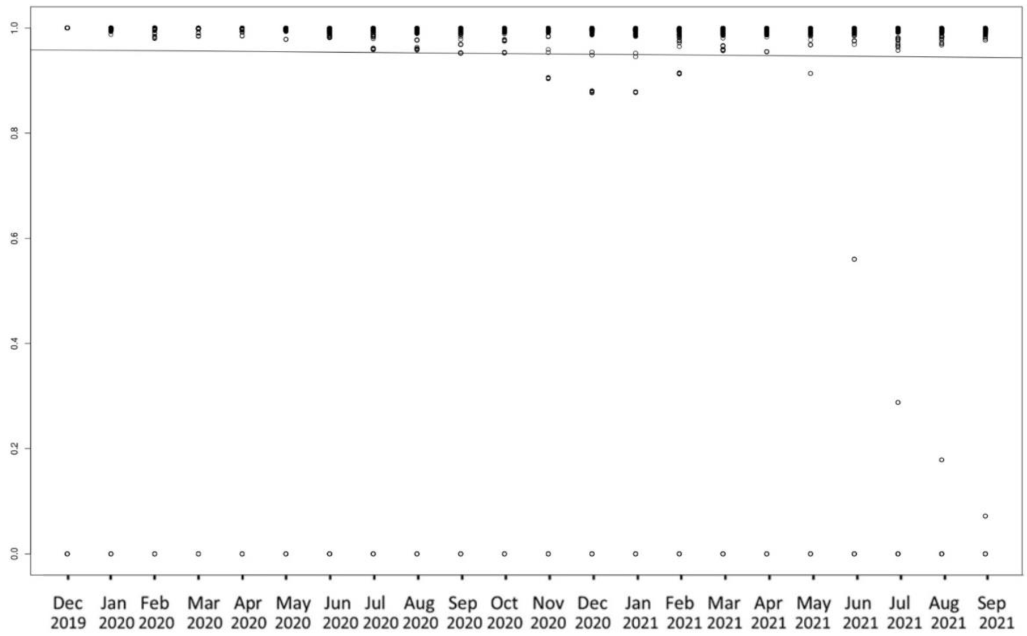

Figure S15: Graph showing linear regression line in monthly conservation of epitopes (BCE) in the NSP14 protein.

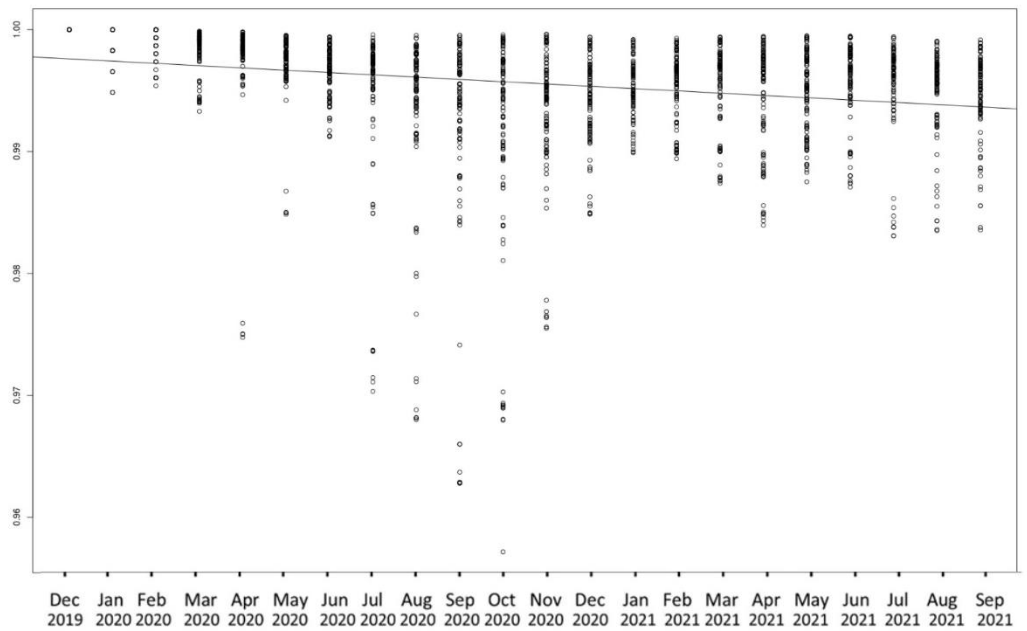

Figure S16: Graph showing linear regression line in monthly conservation of epitopes (BCE) in the NSP15 protein.

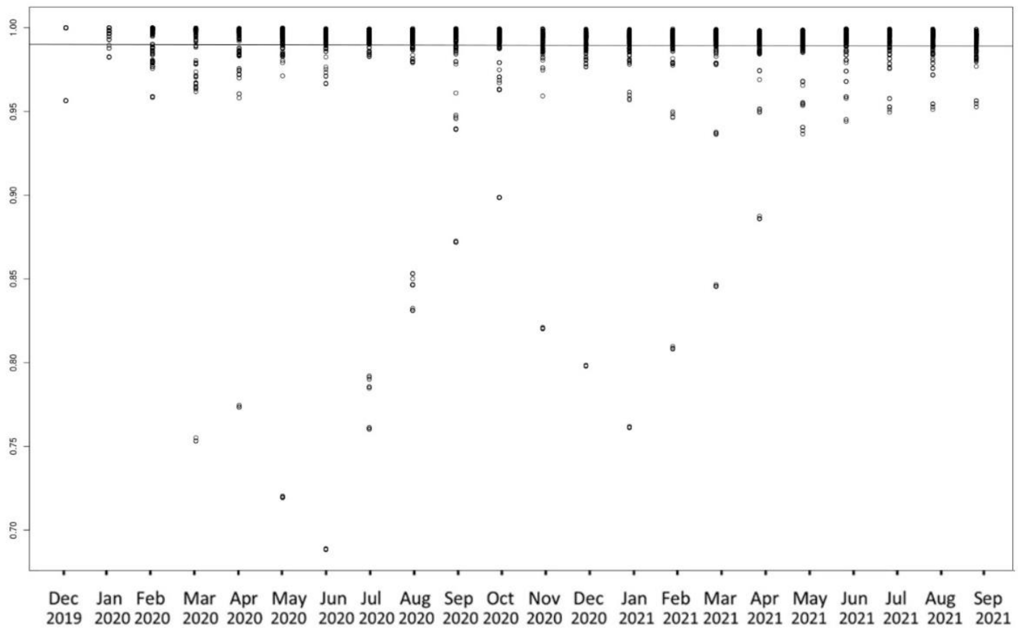

Figure S17: Graph showing linear regression line in monthly conservation of epitopes (BCE) in the NSP2 protein.

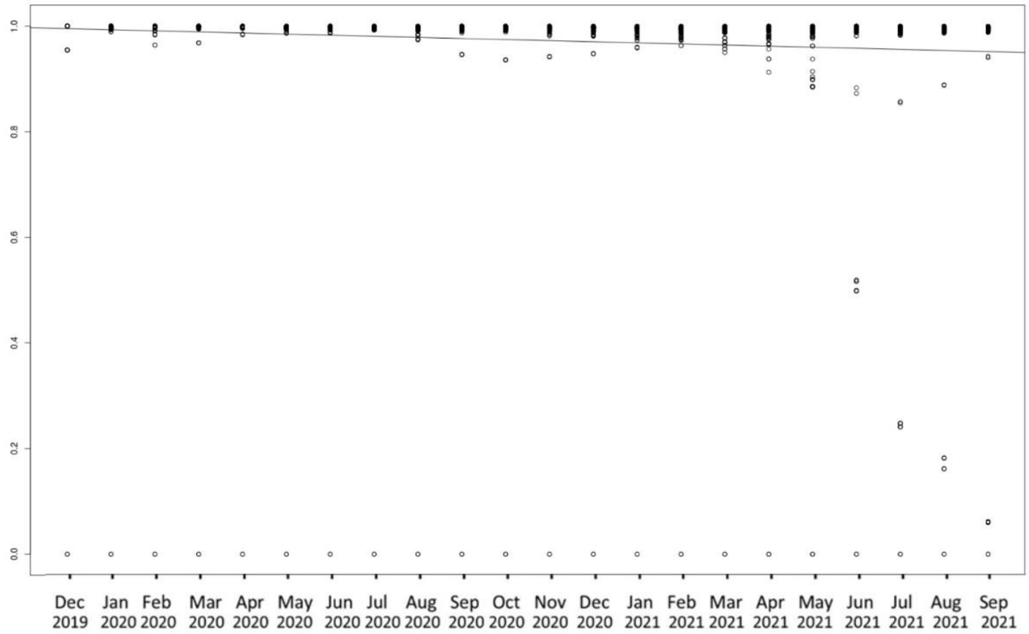

Figure S18: Graph showing linear regression line in monthly conservation of epitopes (BCE) in the NSP4 protein.

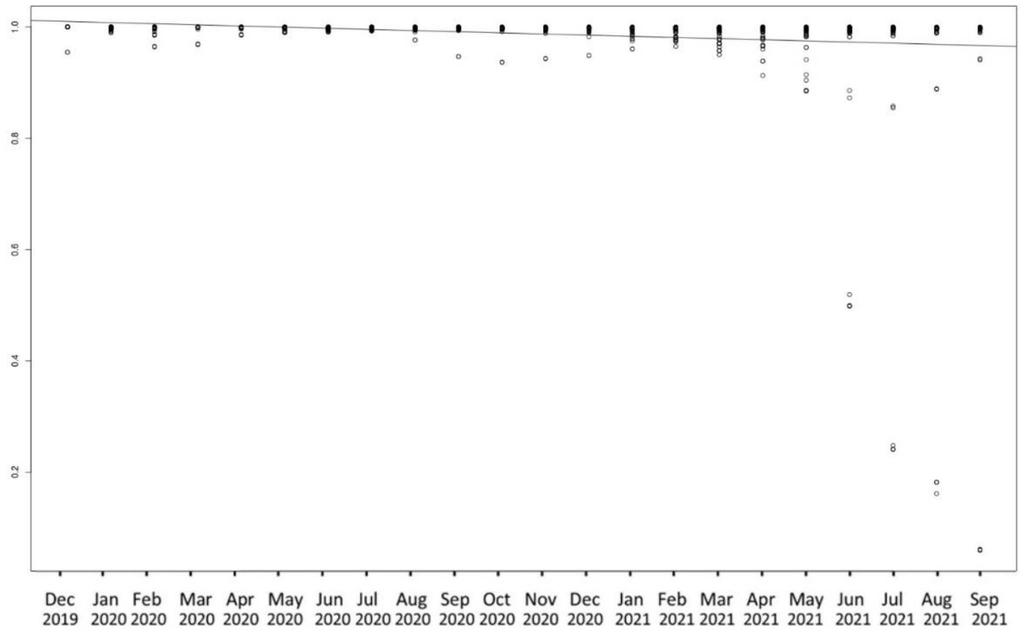

Figure S19: Graph showing linear regression line in monthly conservation of epitopes (TCE) in the NSP4 protein.

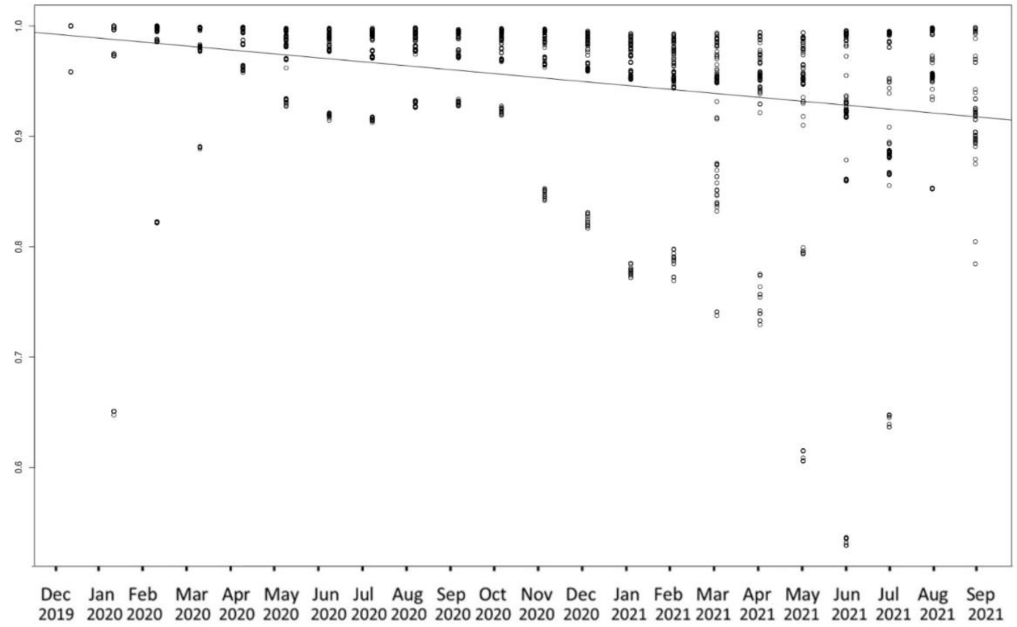

Figure S20: Graph showing linear regression line in monthly conservation of epitopes (BCE) in the NS8 protein.

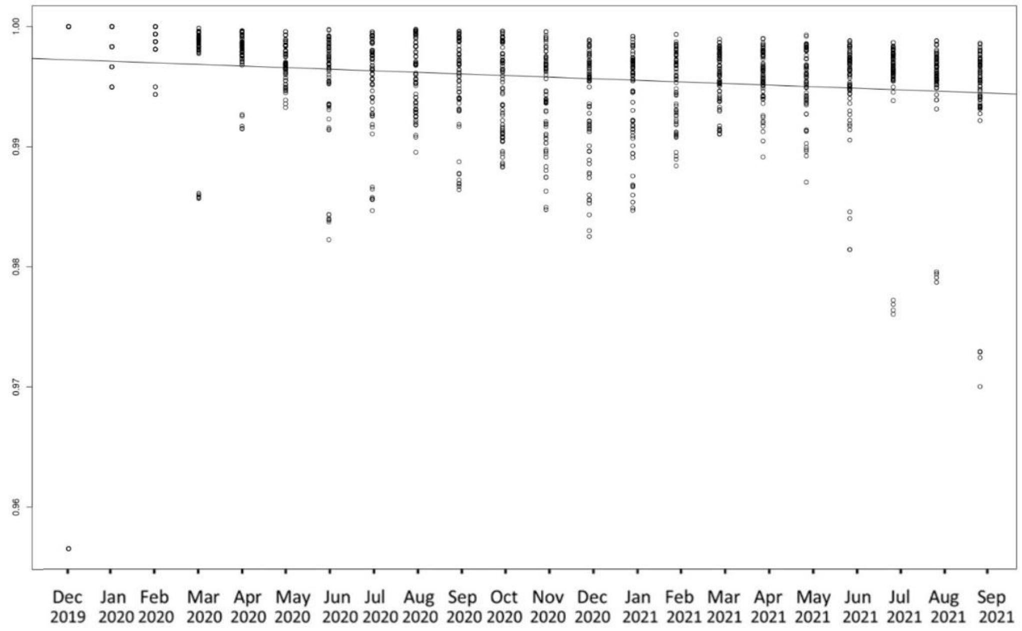

Figure S21: Graph showing linear regression line in monthly conservation of epitopes (BCE) in the NSP1 protein.

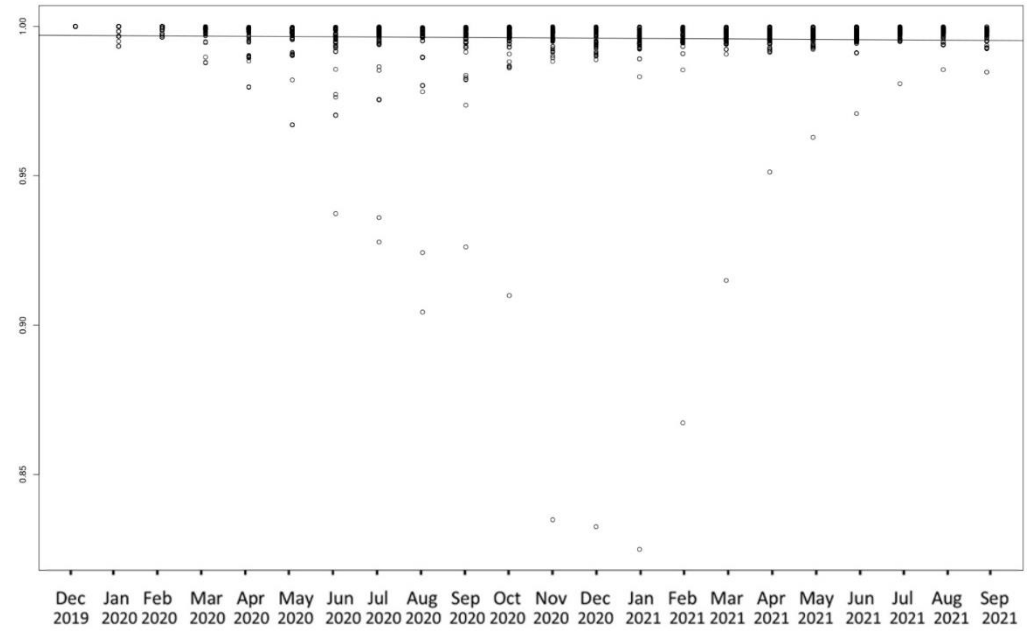

Figure S22: Graph showing linear regression line in monthly conservation of epitopes (BCE) in the NSP5 protein.

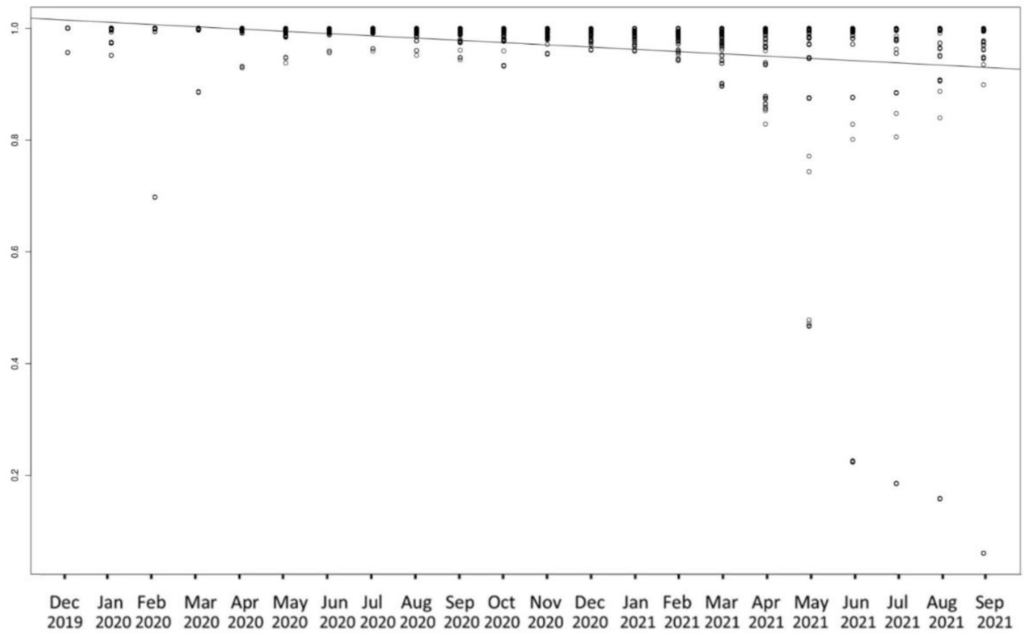

Figure S23: Graph showing linear regression line in monthly conservation of epitopes (BCE) in the NSP6 protein.

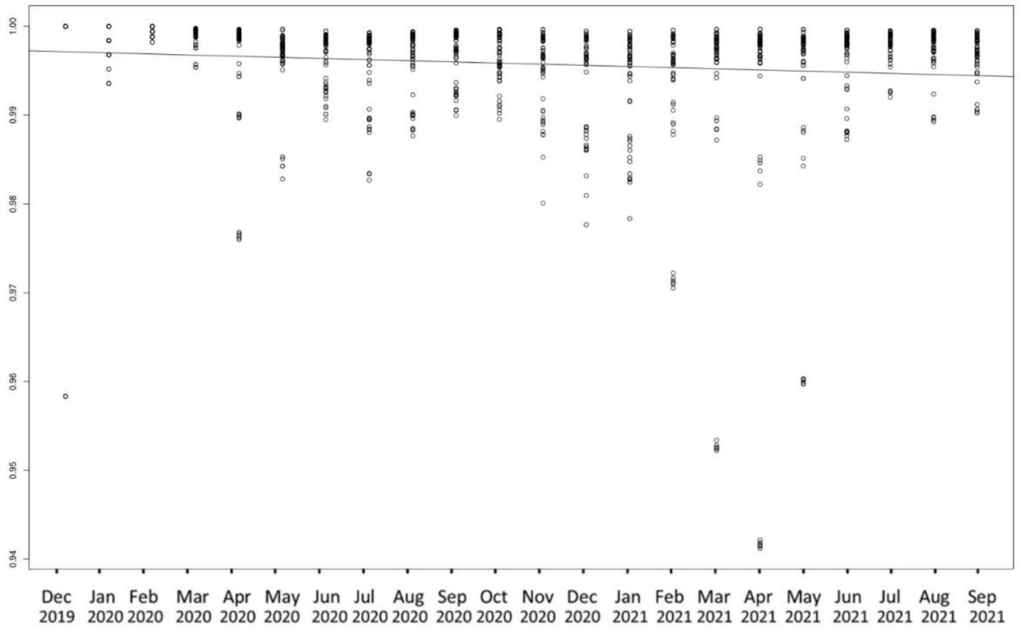

Figure S24: Graph showing linear regression line in monthly conservation of epitopes (BCE) in the NSP8 protein.

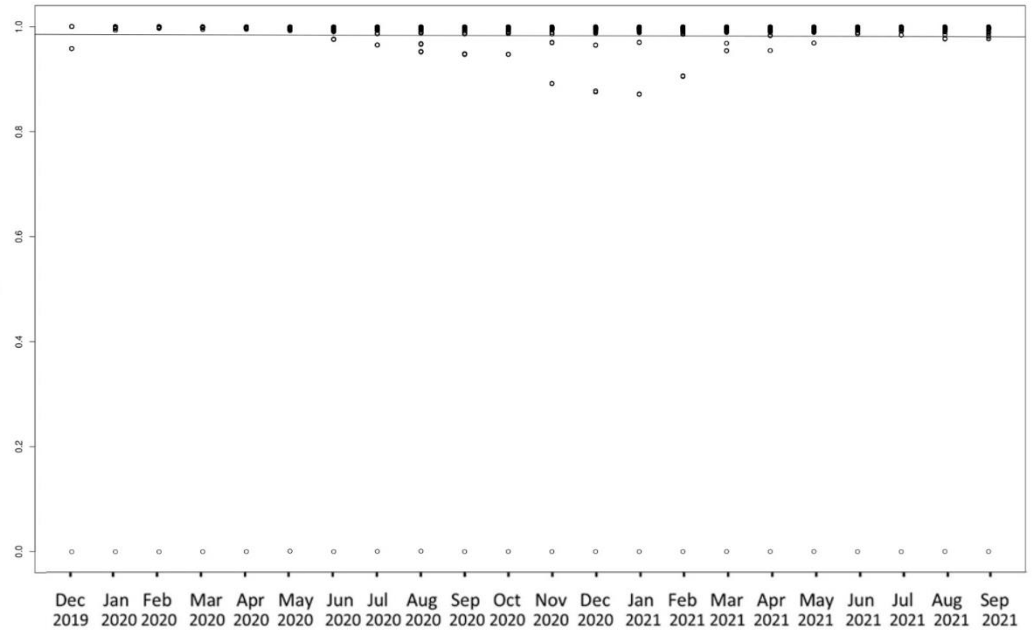

Figure S25: Graph showing linear regression line in monthly conservation of epitopes (BCE) in the NSP16 protein.

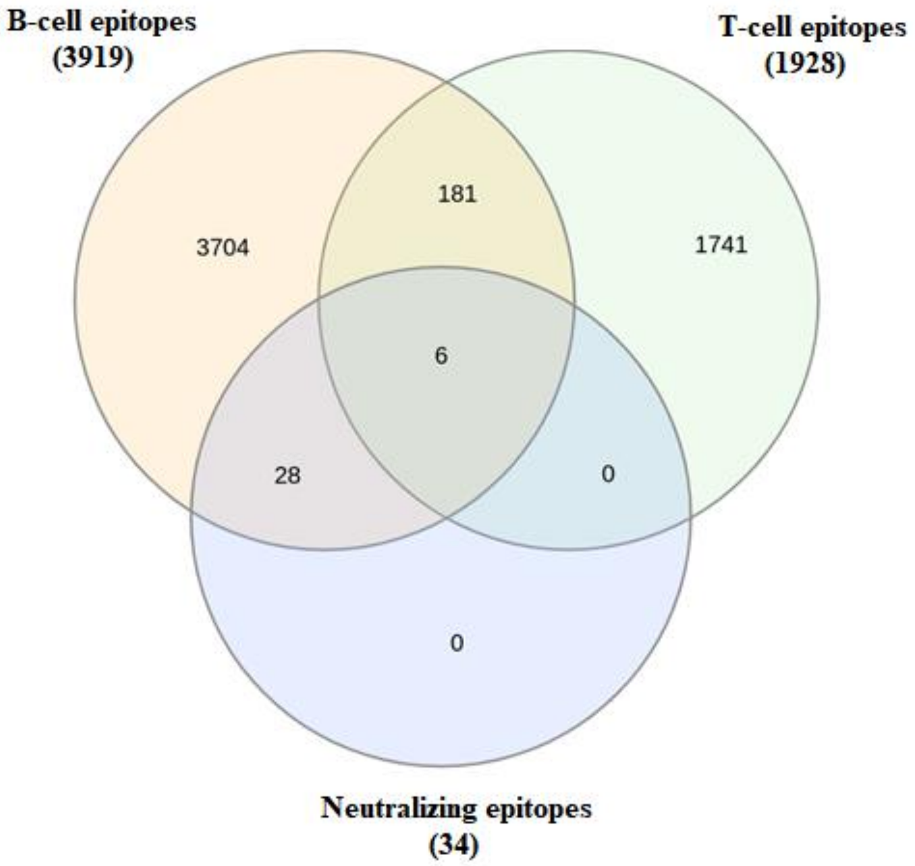

Figure S26: Venn diagram showing common epitopes between neutralizng, B-cell and T-cell epitopes.
